# Dual Antiplatelet Therapy or Antiplatelet Plus Anticoagulant Therapy in Patients with Peripheral and Chronic Coronary Artery Disease: An Updated Review

**DOI:** 10.3390/jcm12165284

**Published:** 2023-08-14

**Authors:** Giulia Magnani, Andrea Denegri, Filippo Luca Gurgoglione, Federico Barocelli, Elia Indrigo, Davide Catellani, Gianluca Signoretta, Alberto Bettella, Domenico Tuttolomondo, Emilia Solinas, Francesco Nicolini, Giampaolo Niccoli, Diego Ardissino

**Affiliations:** 1 Cardiology Division, Parma University Hospital, 43126 Parma, Italy; 2 Cardiac Surgery Division, Parma University Hospital, 43126 Parma, Italy

**Keywords:** coronary artery disease, peripheral artery disease, polyvascular disease, anti-thrombotic therapy, personalized treatment

## Abstract

Despite evidence-based therapies, patients presenting with atherosclerosis involving more than one vascular bed, such as those with peripheral artery disease (PAD) and concomitant coronary artery disease (CAD), constitute a particularly vulnerable group characterized by enhanced residual long-term risk for major adverse cardiac events (MACE), as well as major adverse limb events (MALE). The latter are progressively emerging as a difficult outcome to target, being correlated with increased mortality. Antithrombotic therapy is the mainstay of secondary prevention in both patients with PAD or CAD; however, the optimal intensity of such therapy is still a topic of debate, particularly in the post-acute and long-term setting. Recent well-powered randomized clinical trials (RCTs) have provided data in favor of a more intense antithrombotic therapy, such as prolonged dual antiplatelet therapy (DAPT) with aspirin and a P2Y12 inhibitor or a therapy with aspirin combined with an anticoagulant drug. Both approaches increase bleeding and selection of patients is a key issue. The aim of this review is, therefore, to discuss and summarize the most up-to-date available evidence for different strategies of anti-thrombotic therapies in patients with chronic PAD and CAD, particularly focusing on studies enrolling patients with both types of atherosclerotic disease and comparing a higher- versus a lower-intensity antithrombotic strategy. The final objective is to identify the optimal tailored approach in this setting, to achieve the greatest cardiovascular benefit and improve precision medicine.

## 1. Introduction

**Figure 1:**
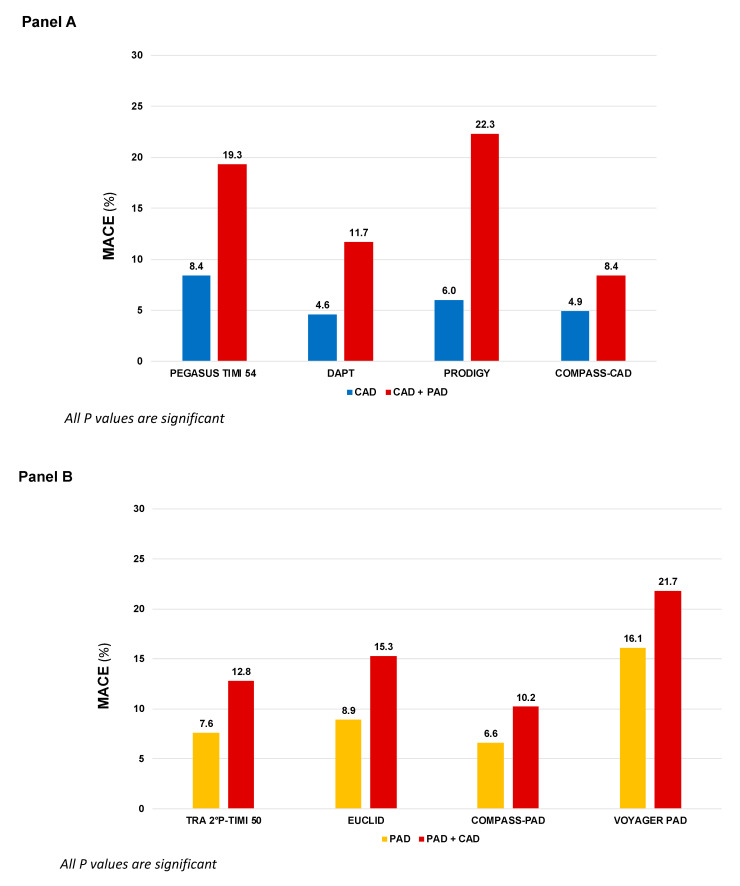
MACE incidence according to different antithrombotic strategies in patients randomized in large clinical trials primarily based on coronary artery disease (**Panel A**) or peripheral artery disease (**Panel B**). COMPASS: Cardiovascular Outcomes for People Using Anticoagulation Strategies; CAD: Coronary artery disease; PAD: Peripheral artery disease; DAPT: Dual antiplatelet therapy; EUCLID: Examining Use of Ticagrelor in Peripheral Artery Disease; PEGASUS-TIMI 54: Prevention of Cardiovascular Events in Patients with Prior Heart Attack Using Ticagrelor Compared to Placebo on a Background of Aspirin–Thrombolysis in Myocardial Infarction 54; TRA 2P–TIMI 50: Thrombin Receptor Antagonist in Secondary Prevention of Atherothrombotic Ischemic Events—Thrombolysis in Myocardial Infarction 50; PRODIGY: Prolonging Dual Antiplatelet Treatment After Grading Stent-Induced Intimal Hyperplasia Study; VOYAGER-PAD: Vascular Outcomes Study of ASA (acetylsalicylic acid) Along with Rivaroxaban in Endo-vascular or Surgical Limb Revascularization for PAD.

Atherosclerosis is a lipid-driven, chronic ubiquitous inflammatory disease of the arterial wall that leads to a broad spectrum of cardiovascular (CV) diseases [1]. Given the systemic nature of the disease, the involvement of more than one vascular bed, known as polyvascular disease, is frequent. Data from the REACH registry showed that approximately 10% of patients with coronary artery disease (CAD) present concomitant peripheral artery disease (PAD) and, vice versa, 60% of patients with PAD present concomitant CAD [2]. In particular, PAD has been associated with more extensity and severity of CAD, including left main disease or more complex CAD [3]. Data from large randomized clinical trials consistently showed that the co-existence of CAD and PAD is a particularly harmful association, both in patients primarily randomized for CAD (Figure 1, Panel A) or PAD (Figure 1, Panel B), with a heightened risk of major adverse cardiovascular events (MACE), including myocardial infarction (MI), stroke and CV death, compared to CAD or PAD only [4]. In addition to an elevated risk of MACE, patients with PAD often require peripheral revascularization and lower extremity amputation, which are associated with increased morbidity and mortality [5].

Beyond controlling modifiable CV risk factors, antithrombotic therapy is the mainstay of secondary prevention, both in chronic PAD and chronic coronary syndrome (CCS). However, the optimal antithrombotic strategy in terms of duration and intensity is still debated, particularly in the long term. Recent evidence supported a long-term dual antiplatelet therapy strategy (DAPT) with aspirin and a P2Y12 or an approach combing an antiplatelet agent and an anticoagulant drug, at the cost of increased risk of bleeding [6,7,8]. 

The aim of this review is, therefore, to discuss and summarize the most updated available evidence derived from large RCTs, investigating different strategies of anti-thrombotic therapies in patients with chronic CAD and PAD (Table 1), in order to identify the optimal tailored approach to achieve the greatest CV benefit and improving precision medicine.

## 2. Recognizing the Burden of Polyvascular Disease as a Chance to Improve Precision Medicine 

Until recently, evidence of efficacy and safety of antithrombotic therapy in patients with polyvascular disease have been derived indirectly from subgroups analyses of studies mainly randomizing patients with CAD. Recent evidence showed, on the other hand, how this population is heterogenous, and how the term PAD, traditionally referring to atherosclerosis in lower extremities, encompasses a larger number of atherosclerotic phenotypes, including carotid, renal arteries and abdominal aortic disease [24]. Interestingly, the presence and extent of carotid plaque was associated long-term with incident coronary artery calcium, even among asymptomatic subjects [25], and a significant correlation between radial and coronary artery calcification has been also found, particularly in patients who underwent coronary revascularization [26]. Similarly, as highlighted in a recent consensus document from EAPCI, the term chronic coronary syndrome (CCS) includes multiple atherosclerotic scenarios, such as patients who never experienced a prior acute coronary syndrome (ACS), as well as those with stabilized CCS at 12 months after ACS [27]. 

This evidence highlights the concept of atherothrombotic disease as a systemic chronic progressive condition, involving multiple artery beds, and representing a CV disease continuum. Traditional CV risk factors such as smoke, hypertension, dyslipidemia, and diabetes are strongly associated with polyvascular disease, suggesting an interplay between multiple common pathways as basis for atherothrombotic progression or destabilization in this setting [28]. Indeed, despite the development of pharmacologic therapies effectively reducing low-density lipoprotein cholesterol, the risk of recurrent CV events in polyvascular disease is still high, supporting the need to target simultaneously other pathways of residual CV risks [29]. For instance, more recent data suggest that patients with polyvascular disease have higher circulating concentrations of inflammatory markers, including interleukin-6 and high-sensitivity C-reactive protein, which is associated to endothelial dysfunction, and platelets hyperreactivity with an overall hypercoagulability state [30]. 

In this regard, polyvascular disease is progressively considered as a marker to discriminate patients at very-high CV risk, and, similar to diabetes, is an independent predictor of long-term CV events recurrence. When the two conditions coexist, the pro-thrombotic pathways, involved in plaque destabilization, are enhanced in a vicious circle [31], and large randomized clinical trials showed that the concomitant presence of polyvascular disease and diabetes exerts a synergistic power in risk prediction, identifying a population with increased long-term CV risk, which, by nature of its higher risk, derives the greatest absolute benefits from aggressive secondary preventive strategies [32]. These observations are confirmed by data from the real-world that showed a worse outcome for patients who underwent a carotid artery stenting if diabetic [33].

Overall, the prompt recognition of the malignant phenotype polyvascular disease, whose associated untoward CV risk is further enhanced by diabetes, is a key issue in order to provide an individualized risk stratification, to consider more aggressive secondary preventive strategies, including a more potent antithrombotic approach. 

## 3. Antithrombotic Therapy in the Chronic Phase of the Disease

### 3.1. Single Antiplatelet Strategy

Single antiplatelet therapy with aspirin is still the most frequently used antiplatelet drug in both chronic CAD and PAD, with a class I recommendation, level of evidence A, in secondary prevention of CCS and in both symptomatic carotid and lower-extremity PAD (LE-PAD) patients [6,7,11,34]. Clopidogrel monotherapy has been studied in the Clopidogrel versus Aspirin in Patients at Risk of Ischemic Events (CAPRIE) enrolling a broad population with stable atherosclerotic cardiovascular disease (ASCVD). In this trial, clopidogrel monotherapy reduced ischemic events compared to aspirin monotherapy [9]. In a post hoc analysis of patients with symptomatic PAD, clopidogrel was associated with an additional one fourth risk reduction in MACE compared to aspirin (HR 0.76, 95% CI: 0.64–0.91, *p* = 0.003) [35] and the European PAD guidelines suggest clopidogrel as the preferred antiplatelet drug (Class IIb, Level of evidence C), whereas the ACC/AHA guidelines did not make a preference statement between agents [7,8]. Although the efficacy of clopidogrel has been confirmed in an extensive meta-analysis including PAD patients who underwent revascularization [36], more recent evidence did not corroborate the superiority of clopidogrel over aspirin as SAPT in PAD patients [37]. This may be partially traced back to the so called “antiplatelet non-responsiveness”, a condition of high on-treatment platelet reactivity present in up to 50% of patients with critical limb ischemia and associated to a significantly increased CV events risk [38]. More recently, in the Examining Use of Ticagrelor in Peripheral Artery Disease (EUCLID) trial, enrolling 13,185 patients with symptomatic PAD, more potent P2Y12 inhibition with ticagrelor monotherapy, did not reduce MACE (HR 1.02, 95% CI: 0.92–1.13, *p* = 0.65) or lower-limb revascularization (HR 0.95, 95% CI: 0.87–1.05, *p* = 0.30) compared to clopidogrel, with a similar rate of major bleeding (HR 1.10, 95% CI: 0.84–1.43, *p* = 0.49) [10]. The use of SAPT with ticagrelor may, therefore, be reserved to PAD patients who are non-responders to clopidogrel. Although neutral, the EUCLID trial is of clinical utility, raising awareness of a higher CV risk in the subgroup of PAD patients with concomitant CAD (*n* = 4032, 29%), that had higher composite rates of CV death, MI, and ischemic stroke (15.3% vs. 8.9%, HR 1.50, 95% CI: 1.13–1.99; *p* = 0.005) compared to those without CAD (Figure 1, Panel B). 

### 3.2. Dual Antiplatelet Therapy 

Aspirin, through the inhibition of the cyclooxygenase 1, and prasugrel, ticagrelor or clopidogrel, blocking the platelet receptor for adenosine diphosphate P2Y12, reduce the production of thromboxane A2 and inhibit platelets activation and aggregation [39]. DAPT is the standard therapy in the acute phase post-ACS; however, duration and type of treatment in patients with stable CAD or PAD is still subject of ongoing debate. 

In the Clopidogrel for High Atherothrombotic Risk and Ischemic Stabilization Management and Avoidance (CHARISMA) trial, 15,603 patients with established CV disease or high-risk profile were randomly assigned either to DAPT with aspirin and clopidogrel or aspirin alone. Although the overall trial was neutral, in the subgroup of patients with prior MI, the addition of clopidogrel to aspirin significantly reduced the primary endpoint of CV death, MI, or stroke (HR 0.77, 95% CI: 0.62–0.98; *p* = 0.03) [12]. In LE-PAD patients (*n* = 3096), MACE were not significantly reduced (HR 0.85, 95% CI: 0.66–1.08; *p* = 0.18), and there was a numerically lower rate of peripheral arterial bypass surgery (*p* = 0.07) with similar risk of leg amputation, at the cost of increased moderate bleeding (HR 1.36, 95% CI: 1.03–1.79; *p* = 0.03). Asymptomatic carotid artery disease was an inclusion criterion in 7% of patients enrolled and no benefit was observed between DAPT and SAPT [13]. The use of DAPT with ticagrelor, in patients with prior MI, was examined in the Prevention of Cardiovascular Events TIMI 54 in Patients with Prior Heart Attack Using Ticagrelor Compared to Placebo on a Background of Aspirin-Thrombolysis in Myocardial Infarction 54 (PEGASUS-TIMI 54). Patients with prior MI and PAD present a double CV risk than those with PAD alone (8.4% vs. 19.3%, placebo group, *p* < 0.001, Figure 1, Panel A). The combination of aspirin and ticagrelor 60 mg twice daily (the approved dose) compared to aspirin alone, provided in 1143 patients with concomitant PAD an absolute risk reduction for MACE of 4.1% and a significant reduction in risk of MALE (HR 0.65, 95% CI: 0.44–0.95, *p* = 0.026) with no differences in TIMI major bleeding (HR 1.18, 95% CI: 0.29–4.70; *p* = 0.82) compared to patients without PAD [20]. Subgroup analyses from the Dual Antiplatelet Therapy (DAPT) and Prolonging Dual Antiplatelet Treatment After Grading Stent-Induced Intimal Hyperplasia Study (PRODIGY) trials provided information regarding extended use of DAPT after coronary stenting in patients with PAD. In the DAPT trial, extended DAPT (clopidogrel or prasugrel plus aspirin for 30 vs. 12 months) was associated with consistent ischemic benefit among patients with (HR: 0.63; 95% CI: 0.32–1.22) and without PAD (HR: 0.53; 95% CI: 0.42–0.66, *p* for interaction = 0.63) [16], while in the PRODIGY trial, there was a significant interaction based on PAD. Prolonged versus shorter DAPT duration was associated with a greater reduction in MACE in patients with PAD (16.1% vs. 27.3%; HR, 0.54; 95% CI, 0.31–0.95; *p* = 0.03) compared with those without PAD (9.3% vs. 7.4%; HR, 1.28; 95% CI, 0.92–1.77; *p* = 0.15, *p* for interaction 0.01) [15]. Based on this overall evidence, the current guidelines for CCS recommend a prolonged DAPT regimen in post-MI patients with a high risk of ischemic events, such as those with concomitant PAD, and without high bleeding risk, if they have tolerated the DAPT regimen for 1 year [6].

Finally, the inhibition thrombin-induced platelet aggregation through PAR-1 with vorapaxar has been investigated in the Thrombin Receptor Antagonist in Secondary Prevention of Atherothrombotic Ischemic Events—Thrombolysis in Myocardial Infarction (TRA-2°P-TIMI 50) which enrolled a broad population of patients with ASCVD [17]. Consistently with the other studies, patients with both CAD and PAD presented with higher risk of MACE (12.8% vs. 7.6%). More interestingly, there was significant heterogeneity in the magnitude of benefit of vorapaxar therapy in PAD patients with CAD, that experienced a greater benefit in term of MACE reduction compared to those with PAD alone (number needed to treat 45 vs. 1000) [19]. In the FDA approved population (patients with CAD or PAD without history of stroke/transient ischemic attack), the benefit of vorapaxar was associated with increased bleeding, leading to a class IIB recommendation in the current American Guidelines [40]. The overall findings highlight the clinical importance of identifying subgroups of patients that derive greater benefit from a more intense antithrombotic approach. 

### 3.3. Therapy with Aspirin Combined with an Anticoagulant Drug

Beside platelets activation and aggregation, coagulation is the other key player in thrombus formation. Atherosclerotic plaque rupture exposes tissue factor, which initiates the coagulation cascade and triggers, through the activation of factor X to factor Xa, thrombin generation and fibrin formation. Both thrombin and factor Xa, through the protease-activated receptors (PARs), enhanced platelet activation, with a continuous cross-talking between the coagulation and platelet pathway. Furthermore, is now well established that in patients with acute coronary syndromes, a hypercoagulable state persists for a prolonged period after clinical stabilization and it is associated with worse CV outcome [41,42]. Finally, factor Xa and thrombin modulate a number of inflammation pathways, which further supports their contributing role in atherogenesis and its thrombotic complications [43]. 

Thus, hypothesizing a synergistic effect, a strategy combining an oral anticoagulant on top of a single antiplatelet agent has been investigated in studies enrolling patients with ASCVD. In particular, the combination of aspirin with a low-dose of the selective direct factor Xa inhibitor rivaroxaban at the dosage of 2.5 mg b.i.d. was shown to reduce the rates of CV death and all-cause death (2.9% vs. 4.5%; HR, 0.68; 95% CI, 0.53–0.87) compared with placebo in the ATLAS ACS 2-TIMI 51 trial (Anti-Xa Therapy to Lower Cardiovascular Events in Addition to Standard Therapy in Subjects with Acute Coronary Syndrome 2–Thrombolysis in Myocardial Infarction 51), enrolling 15 526 patients with acute coronary syndromes [44]. 

This strategy was, therefore, tested in the Cardiovascular Outcomes for People Using Anticoagulation Strategies (COMPASS) trial enrolling patients (*n* = 27,395) with established coronary or peripheral artery disease (included carotid artery disease). Participant subjects have been randomized to rivaroxaban 5 mg OD alone, rivaroxaban 2.5 mg BID plus aspirin and aspirin alone. The trial has been stopped earlier owing to overwhelming proof of efficacy of rivaroxaban 2.5 mg BID plus aspirin, which provided a 24% relative risk reduction in MACE (rivaroxaban plus aspirin vs. aspirin alone: HR 0.76, 95% CI 0.66–0.86, *p* < 0.001; rivaroxaban versus aspirin: HR 0.90, 95% CI 0.79–1.03, *p* = 0.12) driven by reductions in the rate of CV death and stroke. All causes of death were significantly reduced by 18% in the rivaroxaban 2.5 mg plus aspirin arm (HR 0.82, 95% CI 0.71–0.96; *p* = 0.01). Major bleeding, according to a modified International Society on Thrombosis and Haemostasis (ISTH) classification, occurred more frequently in patients in the rivaroxaban plus aspirin group than in those receiving aspirin alone (3.1% vs. 1.9%; HR 1.70, 95% CI 1.40–2.05, *p* < 0.001), driven by gastrointestinal bleeding, with no significant differences in fatal bleeding or intracranial bleeding [21]. 

A subgroup analysis of 6391 patients with LE-PAD demonstrated that aspirin plus a low-dose rivaroxaban, compared to aspirin alone, reduced peripheral vascular outcome by 24% (5.5% vs. 7.1%; *p* = 0.03), MALE by 43% (1.5% vs. 2.6%; *p* = 0.01) and amputation by 58% (0.5% vs. 1.2%; *p* = 0.01), at the cost of a significant increase in major bleeding (2.0% vs. 3.2%; *p* = 0.02), without difference in intracranial or fatal bleeding [5]. The consensus document from the ESC working group on aorta and peripheral vascular diseases recommends, therefore, rivaroxaban 2.5 mg bid (the so-called “vascular-dose”) on top of low-dose aspirin in stable patients with chronic symptomatic PAD without conditions at high risk of bleeding. 

### 3.4. Antithrombotic Therapy after Revascularization 

#### 3.4.1. Lower Extremity Peripheral Revascularization 

After endovascular and surgical lower extremity revascularization, the risk of limb events, particularly acute limb ischemia and amputation, remains high. However, until recently, few powered clinical trials of antithrombotic therapies in this setting have been developed, most of them with poor results. Antiplatelet therapy after endovascular revascularization has often been based on studies of patients undergoing percutaneous coronary interventions (PCI). The European guidelines suggest that DAPT should be considered after infra-inguinal stent implantation for one month (Class IIa, level of evidence C) and the AHA/ACC guidelines state that DAPT (aspirin and clopidogrel) may be reasonable to reduce the risk of limb-related events in patients with symptomatic PAD after lower extremity revascularization (level of evidence C). At the same time, based on the CHARISMA trial [13], the AHA/ACC guidelines give a Class-IIb recommendation for long-term DAPT. In the Management of peripheral arterial interventions with mono or dual antiplatelet therapy (MIRROR) study, a 6-month DAPT strategy with aspirin plus clopidogrel vs. aspirin monotherapy showed, in 80 patients, a lower rate of target lesion revascularization at 6 months (5% vs. 8%, *p* = 0.04), but not at 1 year (25% vs. 32%, *p* = 0.35). No significant differences were observed in terms of bleeding at 6 months (2.5% vs. 5.0%, *p* = ns) [45]. 

After open surgery, the guidelines recommend long-term SAPT. The Clopidogrel and acetylsalicylic acid in bypass surgery for peripheral arterial disease (CASPAR) trial showed no benefit of DAPT with aspirin and clopidogrel versus aspirin alone after lower extremity bypass, with excess of bleeding [46]. The Edoxaban Plus Aspirin vs. Dual Antiplatelet Therapy in Endovascular Treatment of Patients With Peripheral Artery Disease (ePAD) trial compared edoxaban plus aspirin to DAPT with low-dose aspirin and clopidogrel for a period of 3 months after femoro-popliteal endovascular revascularization. The trial was not powered for efficacy, and the risk of restenosis was not different between the two groups (HR 0.89; 95% CI 0.59–1.34). No significant excess in TIMI bleeding was observed with edoxaban (HR 0.56; 95% CI 0.19–1.62) [47]. The Dutch Bypass Oral anticoagulants or Aspirin (DUTCH-BOA) study compared ASA with VKA after infrainguinal bypass surgery, showing no difference for graft patency or MALE with an increased risk of major bleeding, included hemorrhagic stroke [48]. 

The most recent Vascular Outcomes Study of ASA (acetylsalicylic acid) Along with Rivaroxaban in Endovascular or Surgical Limb Revascularization for PAD (peripheral artery disease) (VOYAGER-PAD) is, so far, the larger, more well-powered randomized clinical trial in the peripheral revascularization setting. Overall, 6564 patients who underwent infrainguinal percutaneous or surgical revascularization were enrolled and randomized within 10 days after revascularization to rivaroxaban 2.5 mg twice daily plus aspirin or to placebo plus aspirin [23]. Low-dose rivaroxaban plus aspirin, compared to aspirin alone, significantly reduced the primary composite end point of acute limb ischemia, major vascular amputation, myocardial infarction, ischemic stroke, or CV death (HR 0.85, 95% CI 0.76–0.96; *p* = 0.009) without heterogeneity (*p* for interaction = 0.43) between surgical and endovascular LE-revascularization [49]. Therefore, the use of low-dose rivaroxaban plus aspirin is supported irrespective of the type of treatment. Rates of Thrombolysis in Myocardial Infarction (TIMI) major bleeding were not increased with rivaroxaban, whereas there was a significant excess of major bleeding as defined by the International Society on Thrombosis and Haemostasis (ISTH, HR 1.42; 95% CI 1.10–1.84; *p* = 0.007). The guidelines recommend, therefore, rivaroxaban at the “vascular-dose” on top of low-dose aspirin in patients not at high bleeding risk. 

#### 3.4.2. Carotid Revascularization

Antithrombotic therapy is recommended for secondary prevention of carotid artery disease after carotid artery stenting (CAS) and carotid endarterectomy (CEA). DAPT is recommended in patients undergoing CAS for 1 month, based on two small randomized clinical trials that were terminated earlier due to higher 30-days rate of stent thrombosis and neurological events in the aspirin-alone group, compared to the DAPT (aspirin plus clopidogrel) group, without increase of bleeding [50,51]. The optimal duration of DAPT after CAS should be based on the ischemic-bleeding risk balance. For instance, the occurrence of periprocedural ischemic brain lesions on magnetic resonance imaging has been associated with increased risk for recurrent cerebrovascular events and suggest a potential benefit of more aggressive and prolonged antiplatelet therapy in this scenario [52]. However, the ischemic benefit of a prolonged DAPT strategy must be counterbalanced by the potential increased in intracranial hemorrhage, particularly in those patients with recent stroke. DAPT may, therefore, be prolonged beyond 1 month after CAS if low-bleeding risk or in patients with concomitant CAD, particularly if at high ischemic risk, such as patients with a recent MI.

A meta-analysis involving 36,881 patients after CEA and 150 after CAS showed no differences in the composite of stroke, transient ischemic attack or death between SAPT and DAPT after CEA, but there was a significant risk of major bleeding and neck hematoma with DAPT [53]. The current guidelines and consensus document recommend, therefore, at least 1-month DAPT after CAS and life-long SAPT after CEA [6,7].

#### 3.4.3. Coronary Revascularization in Chronic Coronary Syndrome

A recent consensus document recognized the heterogeneity of the population of CCS patients, recommending the stratification of patients based on the presence or absence of a previous MI and on different revascularization strategies. Specifically for patients without a previous MI who underwent a PCI, the evidence for the optimal antithrombotic strategy emerged from RCTs, comparing a shorter versus a longer DAPT regimen, that often enrolled a mixed population represented by both CCS and ACS patients. Overall, the benefit of a prolonged DAPT duration in terms of MACE reduction was counterbalanced by an increase in bleeding, which tended to neutralize the net benefit [54]. Particularly in high bleeding risk patients, a recent meta-analysis of 11 randomized clinical trials involving 9006 patients at high bleeding risk undergoing PCI, a shorter DAPT regimen (1–3 months) was associated with lower bleeding and CV mortality, without increasing ischemic events, compared with a longer (≥6-months) DAPT regimen [55]. Therefore, also in this setting, the balance between ischemic and bleeding risk is a key issue. After PCI, 1- to 6-month DAPT, followed by long-life SAPT monotherapy is the default approach; however, patients at higher bleeding risk may benefit from shortening DAPT with aspirin and clopidogrel to 1 to 3 months with transition to SAPT with either ASA or a P2Y12 receptor inhibitor. On the other hand, in patients with a prevalent ischemic risk, the intensity of the antithrombotic therapy can be increased with a prolonged DAPT duration (>6 months) or by adding to aspirin a low-dose rivaroxaban. The latter strategy is particularly recommended in patients at low bleeding risk and at high risk for stroke, including patients with concomitant PAD [56]. For patients who underwent a coronary artery bypass grafting (CABG), a SAPT strategy with aspirin or clopidogrel is recommended. In the prespecified subgroup analysis form the COMPASS trial in CABG patients, aspirin plus rivaroxaban 2.5 mg did not affect graft patency compared with aspirin alone, with a consistent benefit in MACE as seen in the overall population [57]. Therefore, similarly to patients undergoing PCI, this strategy may be considered when in patients in whom concerns over ischemic events prevail over bleeding, precisely as patients with polyvascular disease. 

To further improve personalization in antithrombotic approaches, bleeding and ischemic risk should be integrated with the individual responsiveness to an antiplatelet agent [54]. On-treatment platelet reactivity assessed by platelet function test or genetic testing in order to assess genetic polymorphisms of the hepatic cytochrome P450 (CYP) system in patients undergoing PCI to guide the selection of P2Y12 inhibitor may be useful to inform the clinician to either escalation (from more potent to less potent P2Y12 inhibitor) or de-escalation of therapy (from more potent P2Y12 inhibitor to clopidogrel), in order to reduce interindividual variability and consequently bleeding and thrombotic complications [58].

## 4. Primary Prevention

If the role of antithrombotic therapy in secondary prevention of patients with PAD or CAD is well established, its efficacy–safety balance in primary prevention is more questionable and controversial. The marginal net benefit of aspirin has been confirmed in the most contemporary trials conducted in primary prevention of higher CV risk patients. For instance, in The Aspirin in Reducing Events in the Elderly (ASPREE) [59], in both the ASCEND (A Study of Cardiovascular Events iN Diabetes) [60] and Aspirin and simvastatin combination for cardiovascular Events Prevention Trial in Diabetes (ACCEPT-D) [61] trials, and in the Aspirin to reduce risk of initial vascular events (ARRIVE) trial, which examined patients with a moderate risk of a 10-year CV event [62], the net benefit of aspirin was marginal with increased a major bleeding. 

Furthermore, it is uncertain whether aspirin provides net benefit in asymptomatic patients with incidental detection of non-obstructive CAD or increasing coronary artery calcium on imaging which was associated with both bleeding and ischemic hazard [27,63]. The current guidelines state that SAPT in primary prevention may be considered in patients at very high risk of CV events, such as patients with a 10-year CV risk of 10%. 

Similarly, data regarding antiplatelet therapy in asymptomatic PAD patients are conflicting. In asymptomatic patients with a significant (≥50%) carotid stenosis, ESC guidelines recommend (Class IIa) SAPT with aspirin or clopidogrel in patients at low-bleeding risk, whereas the routine use of SAPT is not recommended (Class III) in asymptomatic LE-PAD unless concomitant another indication, such as CAD [7]. On the contrary, ACA/AHA guidelines acknowledge SAPT as a reasonable option in asymptomatic patients with ankle brachial index (ABI) ≤ 0.90, for MACE reduction [8]. In the Prevention of Progression of Arterial Disease And Diabetes (POPADAD) study, aspirin did not show a benefit compared to placebo (HR 0.88, 95% CI 0.76–1.26) in primary prevention of diabetic patients [64]. Similarly, in the Aspirin for Asymptomatic Atherosclerosis (AAA) trial enrolling patients with an ABI ≤ 0.95, aspirin did not reduce MACE (HR 1.03, 95% CI 0.84–1.27) and increased bleeding (HR 1.71, 95% CI 0.99–2.97) [65]. Considering the high proportion of patients with PAD and subclinical CAD, SAPT may be a reasonable option in patients with asymptomatic PAD, impaired ABI and with low bleeding risk. 

### A Practical Approach

**Figure 2:**
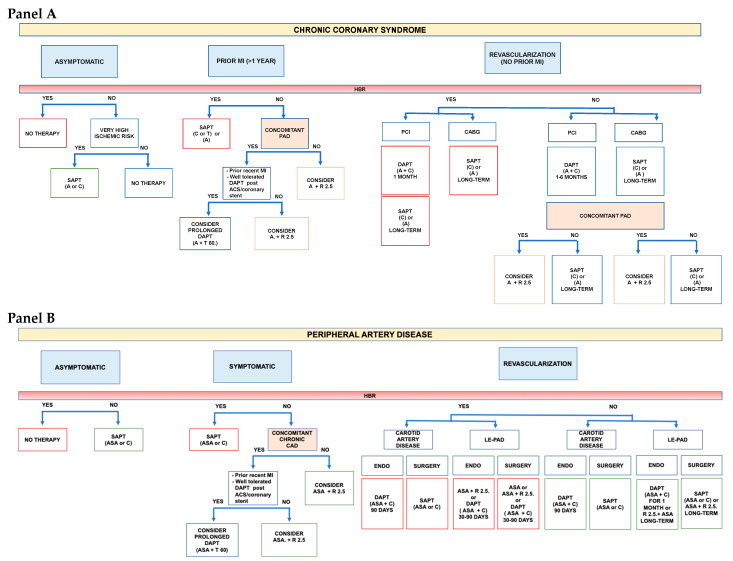
Selecting antithrombotic therapy in patients with CCS (**Panel A**) and PAD (**Panel B**): a practical approach. High bleeding risk (HBR) defined as history of intracerebral hemorrhage or ischemic stroke, history of other intracranial pathology, recent gastrointestinal bleeding or anemia due to possible gastrointestinal blood loss, other gastrointestinal pathology associated with increased bleeding risk, liver failure, bleeding diathesis or coagulopathy, extreme old age or frailty, or renal failure requiring dialysis or with eGFR < 15 mL/min/1.73 m^2^. CCS: Chronic coronary syndrome; PAD: peripheral artery disease; DAPT: Dual antiplatelet therapy; SAPT: Single antiplatelet therapy; R: Rivaroxaban; C: Clopidogrel; T: Ticagrelor; ASA: Aspirin; MI: Myocardial infarction; ACS: Acute coronary syndrome; PCI: Percutaneous coronary intervention; CABG: Coronary artery bypass graft; ENDO: Endovascular.

Although antithrombotic therapy is the cornerstone of both chronic CAD and PAD, the optimal strategy is still debated, particularly when these conditions are associated. Available evidence suggests that the higher the burden of atherosclerosis, the higher the benefit of a high intensity antithrombotic therapy. The price to be paid is an increase in major bleeding, primarily of gastrointestinal origin, without excess in intracranial or fatal bleeding. Therefore, in accordance with the most recent ESC guidelines [6], the first step for a practical approach in selecting the optimal antithrombotic in patients with stable CAD or PAD, is the evaluation of the bleeding risk (Figure 2). Only patients at low bleeding risk should be considered for a regimen of high intensity antithrombotic therapy. In this contest, two factors, anemia at baseline and a previous history of bleeding requiring hospitalization, have emerged as useful characteristic to identify patients at increased risk of bleeding [66]. Those patients should be considered for SAPT, always taking into account that bleeding risk changes over time and periodic re-assessment is mandatory [67]. 

For patients at low bleeding risk, the following step is the evaluation of the ischemic risk. Patients with CAD, especially with multivessel disease, and concomitant PAD have been categorized to be at high ischemic risk and a more intense antithrombotic regimen with DAPT or DAT may, therefore, be considered in this group. The coexistence of diabetes represents a particular malignant phenotype, which warrants a particularly aggressive secondary preventive strategy and a strict follow up. In patients with CAD or PAD, who experienced a recent MI and have well-tolerated the “bleeding stress-test” of the 1-year period of DAPT following the acute event, a prolonged DAPT therapy may be considered over a strategy combining aspirin and an anticoagulant. Based on the results of the PEGASUS TIMI 54 trial, ticagrelor 60 mg b.i.d. should be the preferred option [20,67].

In patients with PAD and concomitant CAD, but without a recent ACS, or in PAD patients with a previous peripheral revascularization, a regimen with low dose rivaroxaban 2.5 mg b.i.d. on top of aspirin may be proposed, based on the results of the COMPASS [21] and VOYAGER trial [45], Figure 2. 

## 5. Conclusions

Based on the overall results of RCTs and subgroup analyses of patients with coronary and peripheral artery disease, antithrombotic therapy should be individualized considering the clinical presentation and burden of the atherosclerotic disease, together with the bleeding–ischemic balance. A stepwise selection may be useful for clinicians to identify those patients who benefit the most from a more intense antithrombotic therapy. Further data regarding subgroup of patients with PAD in large RCTs of CAD and, above all, RCTs randomizing primarily patients with PAD are needed in order to further define the optimal antithrombotic therapy in this setting and improving precision medicine. With this final objective, dedicated guidelines for patients with polyvascular disease would be desirable.

## Figures and Tables

**Table 1:** Trials comparing in patients with peripheral artery disease and/or chronic coronary syndrome a higher versus lower intense antithrombotic regimen.

Trial	First Author, Year	Population	Total *n*	Proportion with Both CAD and PAD	Comparison	Median FUP	PEP MACE HR (95% CI)	MAJOR BLEEDING HR (95% CI)
Single antiplatelet therapy (more vs. less potent)								
CAPRIE	CAPRIE Steering Committee, 1996 [9]	Recent MI (*n* = 6302) or Symptomatic LE PAD (*n* = 6452) or Ischemic stroke (*n* = 6431)	19,185	N.A.	Clopidogrel 75 mg vs. Asprin 325	22.9	LE-PAD: 0.76 (0.64–0.91)	Overall population Severe GI bleeding: 0.49% (clopidogrel) vs. 0.71% (aspirin) *p* < 0.05
EUCLID	Hiatt WR, 2017 [10] Berger J, 2018 [11]	Symptomatic LE PAD	13,885	29.0%	Ticagrelor 90 mg b.i.d. vs. Clopidogrel 75 mg	30	1.02 (0.92–1.13)	1.10 (0.84–1.43)
DAPT vs. Single antiplatelet therapy								
CHARISMA	Bhatt DL, 2006 [12] Cacoub P, 2009 [13]	CCS (*n* = 5835) or LE PAD (*n* = 2838) or Ischemic stroke/TIA (*n* = 4290) or multiple atherosclerotic risk factors (*n* = 3284)	15,603	N.A.	Clopidogrel 75 mg + Aspirin 75–162 mg vs. Aspirin 75–162 mg	28	Prior MI: 0.77 (0.62–0.98) LE-PAD: 0.85 (0.66–1.08),	Overall secondary prevention population: 1.10 (0.81–1.54) LE-PAD: 0.97 (0.56–1.66)
PRODIGY	Valgimigli M, 2012 [14] Franzone A, 2016 [15]	CCS (*n* = 505) or ACS (*n* = 1465)	1970	12.5%	Clopidogrel 75 mg + Aspirin 80–160 mg for 24 months vs. 6 months	28	LE-PAD: 0.54 (0.31–0.95) NO-LE-PAD:1.28 (0.92–1.77) *p*-int = 0.01	LE-PAD: 0.76 (0.17–3.40) NO-LE-PAD: 2.07 (1.12–3.83) *p*-int = 0.22
DAPT	Secemsky EA, 2017 [16]	Patients free from ischemic and bleeding events 12 months after coronary stenting	11,648	5.57%	Continued thienopyridine (clopidogrel 75 mg or prasugrel 10 mg) + Aspirin therapy for an additional 18 months vs. Aspirin 100 mg	18	LE-PAD: 0.63 (0.32–1.22) NO-LE-PAD: 0.53 (0.42–0.66) *p*-int = 0.63	LE-PAD: 1.82 (0.87–3.83) NO-LE-PAD: 1.66 (1.23–2.24) *p*-int: 0.81
TRA-2°P-TIMI 50	Bonaca MP, 2012 [17] Magnani G, 2015 [18] Qamar A, 2020 [19]	Stable MI (*n* = 17,779) or LE PAD (*n* = 3787) or Ischemic stroke (*n* = 4883)	26,449	76.3% of 6136 patients with PAD regardless of stratum	Vorapaxar 2.5 mg vs. Placebo	30	PAD + CAD: 0.82 (0.69–0.97) ARD −2.2 (−4.0, −0.4) PAD only: 1.0 (0.69−1.46) ARD 0.1 (−2.6, 2.9)	LE-PAD (overall population): 1.39 (1.12–1.71)
PEGASUS TIMI-54	Bonaca MP, 2016 [20]	Stable MI	21,162	CAD + PAD 5.2%	Ticagrelor 60 or 180 mg b.i.d. + Aspirin 75–150 mg o.d. vs. or Aspirin 75–150 mg o.d.	33	LE-PAD (60 mg): 0.69 (0.47–0.99)	LE-PAD: (dose pooled) 1.32 (0.41–4.20)
Therapy with aspirin combined with an anticoagulant drug								
COMPASS	Eikelboom JW, 2017 [21] Connolly S, 2018 [22] Anand SS, 2018 [5]	Stable CAD (*n* = 24,828) or [Symptomatic LE PAD or carotid artery disease or ABI < 0.9 with CAD (*n* = 7470)]	27,395	COMPASS-CAD: 17.9% COMPASS-PAD:44.1%	Rivaroxaban 2.5 b.i.d. + Aspirin 100 mg or Rivaroxaban 5 mg b.i.d. vs. Aspirin 100 mg	23	Overall population: 0.76 (0.66–0.86)	Overall population: 1.49 (0.67–3.33)
VOYAGER-PAD	Bonaca MP, 2020 [23]	LE PAD with recent peripheral revascularization	6564	PAD + CAD 23.6%	Rivaroxaban 2.5 mg b.i.d. + Aspirin 100 mg vs. Aspirin 100 mg	28	0.85 (0.76–0.96)	1.42 (1.10–1.84)

ACS: acute coronary syndrome; CAPRIE: Clopidogrel Versus Aspirin in Patients at Risk of Ischemic Events artery disease; BID: Twice a day; CAD: Coronary artery disease; CCS: Chronic coronary syndrome; CHARISMA: Clopidogrel for High Atherothrombotic Risk and Ischemic Stabilization, Management, and Avoidance; COMPASS: Cardiovascular Outcomes for People Using Anticoagulation Strategies; DAPT: Dual antiplatelet therapy; EUCLID: Examining Use of Ticagrelor in Peripheral Artery Disease; FUP: Follow-up; LE-PAD: Lower-extremity peripheral artery disease; OD: Once daily; MI: Myocardial infarction; PEGASUS-TIMI 54: Prevention of Cardiovascular Events in Patients with Prior Heart Attack Using Ticagrelor Compared to Placebo on a Background of Aspirin–Thrombolysis in Myocardial Infarction 54; SAPT: Single antiplatelet therapy; TIA. Transient ischemic attack; TRA 2P–TIMI 50: Thrombin Receptor Antagonist in Secondary Prevention of Atherothrombotic Ischemic Events—Thrombolysis in Myocardial Infarction 50; PRODIGY: Prolonging Dual Antiplatelet Treatment After Grading Stent-Induced Intimal Hyperplasia Study; VOYAGER-PAD: Vascular Outcomes Study of ASA (acetylsalicylic acid) Along with Rivaroxaban in Endo-vascular or Surgical Limb Revascularization for PAD; PEP: Primary endpoint; MACE: Major adverse cardiovascular events; HR: Hazard ratio; CI: Confidence intervals; ARD: Absolute risk difference; GI: Gastrointestinal.

## Data Availability

Not applicable.
